# Nutritional Aspects of Iron in Health and Disease

**DOI:** 10.3390/nu15112441

**Published:** 2023-05-24

**Authors:** Edouard Charlebois, Kostas Pantopoulos

**Affiliations:** 1 Lady Davis Institute for Medical Research, Jewish General Hospital, Montreal, QC H3T 1E2, Canada; edouard.charlebois@mail.mcgill.ca; 2 Department of Medicine, McGill University, Montreal, QC H4A 3J1, Canada

**Keywords:** iron deficiency, iron overload, anemia, heme, metabolic syndrome, cardiovascular disease, cancer, microbiome

## Abstract

Dietary iron assimilation is critical for health and essential to prevent iron-deficient states and related comorbidities, such as anemia. The bioavailability of iron is generally low, while its absorption and metabolism are tightly controlled to satisfy metabolic needs and prevent toxicity of excessive iron accumulation. Iron entry into the bloodstream is limited by hepcidin, the iron regulatory hormone. Hepcidin deficiency due to loss-of-function mutations in upstream gene regulators causes hereditary hemochromatosis, an endocrine disorder of iron overload characterized by chronic hyperabsorption of dietary iron, with deleterious clinical complications if untreated. The impact of high dietary iron intake and elevated body iron stores in the general population is not well understood. Herein, we summarize epidemiological data suggesting that a high intake of heme iron, which is abundant in meat products, poses a risk factor for metabolic syndrome pathologies, cardiovascular diseases, and some cancers. We discuss the clinical relevance and potential limitations of data from cohort studies, as well as the need to establish causality and elucidate molecular mechanisms.

## 1. Nutritional Value of Iron

**Figure 1:**
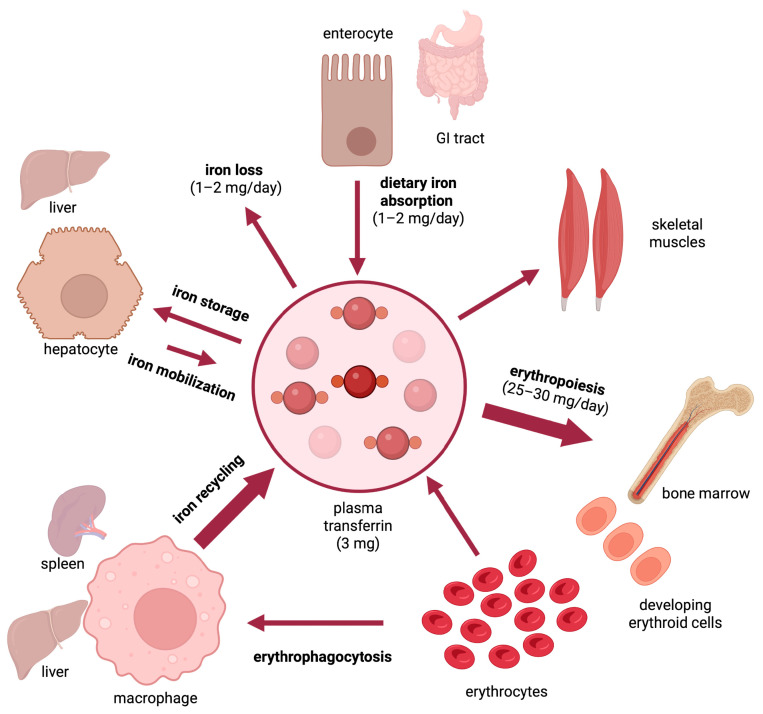
Dynamics of iron traffic in the human body.

Iron is an essential nutrient that is required for critical biological functions such as oxygen transport and cellular respiration. The adult human body contains 3–5 g of iron, with ~70% utilized in hemoglobin of red blood cells [1]. The daily iron requirements for erythropoiesis are 25–30 mg and are mostly met by iron recycled from senescent red blood cells, which are cleared by tissue macrophages (Figure 1). Excess iron is stored in the liver and can be mobilized on demand. As there is no mechanism for iron excretion from the body, dietary absorption of 1–2 mg iron per day is essential to compensate for non-specific iron losses. It should be noted that under physiological conditions, only a small fraction (~15–20%) of ingested luminal iron gets eventually absorbed. This depends on the type of diet, with an estimated 14–18% of iron being absorbed from mixed diets and 5–12% from vegetarian diets [2]. Body iron stores also affect dietary iron absorption. Thus, in iron-deficient individuals with depleted body iron stores and increased iron demand, the maximum absorption for inorganic iron has been reported at 20% and for heme iron at 35% [3].

**Figure 2:**
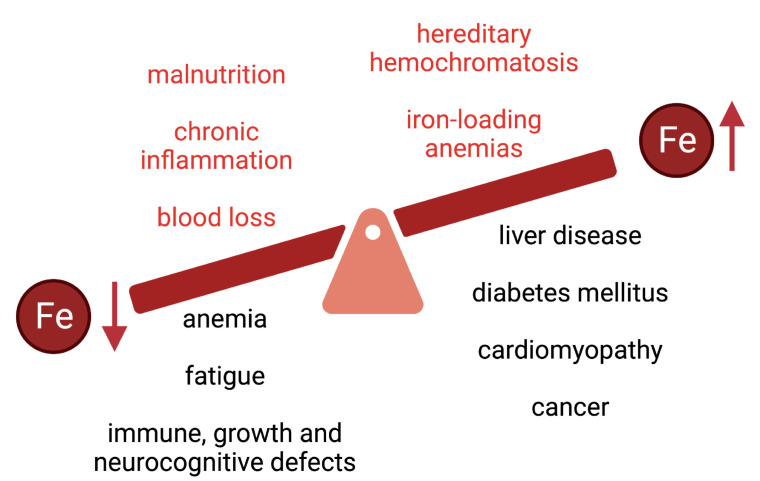
Common causes and clinical complications of iron deficiency and iron overload.

The overall limited bioavailability of iron can lead to iron deficiency anemia or non-anemic iron deficiency, which are the most common pathologies worldwide and remain leading contributors to the global burden of disease (reviewed in [4,5,6]). Iron deficiency is associated with fatigue and may also lead to immune, growth, and neurocognitive defects (Figure 2). In 2005, anemias affected roughly a quarter of the world’s population with iron deficiency anemia accounting for about half of these cases [7]. Little progress has been made since then as, in 2016, iron deficiency anemia was one of the top five causes of years lived with disability with over 1.2 billion cases reported [8].

Nutritional guidelines developed by the Food and Nutrition Board at the National Academy of Medicine in the United States recommend that infants between 7–12 months obtain 11 mg of iron from their diet daily, whereas the corresponding values for adult men, menstruating women and pregnant women are 8, 18 and 27 mg of iron, respectively [9]. These guidelines highlight the elevated iron requirements in pregnant women and infants where iron is critical for growth and development [10].

Iron deficiency and anemia are more prevalent in vulnerable populations such as indigenous peoples, refugees and immigrants from low and middle-income countries, and disadvantaged subpopulations [6,11,12]. These notions provide the rationale for food iron fortification programs [13], and for the use of oral iron supplements or intravenous iron for therapeutic purposes [14]. Nevertheless, excess body iron may lead to adverse health outcomes [15], mainly due to the redox reactivity of the metal that can promote oxidative stress and tissue damage [16]. This is vividly illustrated in diseases of iron overload (Figure 2) such as hereditary hemochromatosis or iron-loading anemias (including thalassemia), which are associated with type 1 and type 2 diabetes mellitus, arthropathy, osteoporosis, hypogonadism, liver disease (fibrosis, cirrhosis, hepatocellular carcinoma) and cardiomyopathy (reviewed in [17,18,19,20,21,22,23,24]). Thus, balanced iron intake is critical to avoid states of iron deficiency or overload.

The hazardous effects of excess iron are also evident in cases of acute iron poisoning, for instance following accidental ingestion of iron supplements by children [25]. This causes gastric and duodenal mucosal necrosis, the severity of which depends on the amount of iron ingested. Symptoms of iron intoxication include nausea, vomiting, diarrhea, gastrointestinal bleeding, coagulopathy, shock, metabolic acidosis, hepatotoxicity, and abdominal pain.

## 2. Iron Homeostasis

**Figure 3:**
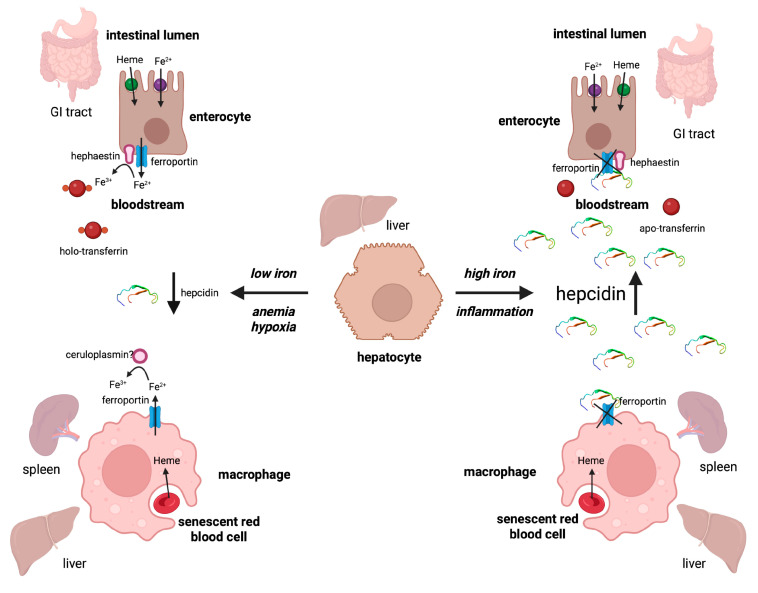
Regulation of systemic iron traffic by hepcidin.

Systemic iron homeostasis is largely controlled by hepcidin, a peptide hormone [26]. Circulating hepcidin is primarily produced by hepatocytes in the liver and targets the iron-exporter ferroportin in intestinal enterocytes, tissue macrophages, and other cells (Figure 3). The binding of hepcidin occludes ferroportin’s iron export channel and, additionally, triggers internalization and lysosomal degradation of ferroportin, limiting iron entry into the bloodstream [27,28,29]. Hepcidin is transcriptionally induced in response to iron or inflammatory cues via BMP/SMAD and IL-6/STAT3 signaling, respectively [30,31]. BMP6 and BMP2 are major hepcidin inducers and are produced by liver sinusoidal endothelial cells. BMP6 is transcriptionally activated by iron and plays a key role in the iron-dependent induction of hepcidin, which serves to prevent excessive dietary iron absorption and systemic iron overload [26]. On the other hand, BMP6 can be neutralized by erythroferrone (ERFE) a bone-marrow-derived hepcidin suppressor that emerges under hypoxemia. Inhibition of hepcidin expression under iron deficiency or hypoxemia allows increased iron absorption and mobilization from stores to meet physiological needs.

Inflammatory induction of hepcidin is considered an innate immune response that causes hypoferremia to deprive extracellular pathogens of iron, which is essential for their proliferation. However, persistent hypoferremia due to chronic inflammatory induction of hepcidin results in functional iron deficiency by restricting iron availability for erythropoiesis, thereby contributing to anemia of inflammation, also known as anemia of chronic disease [32]. Defective iron-dependent regulation of hepcidin due to genetic inactivation of upstream regulators leads to hereditary hemochromatosis, while suppression of hepcidin due to ineffective erythropoiesis contributes to iron overload in thalassemia and other iron-loading anemias [33].

## 3. Iron Biomarkers: Applications and Limitations

The major serum biomarkers typically used to evaluate iron status are ferritin, iron, transferrin saturation, soluble transferrin receptor 1 (sTfR1; mostly referred to as sTfR), and hepcidin [34]. Ferritin is normally an intracellular iron storage protein that can also be found in circulation for reasons that remain unclear. Serum ferritin typically reflects body iron stores but is also influenced by inflammation, liver disease, obesity, and malignancies and must thus be paired with other tests for accurate diagnosis [35,36,37,38]. 

Measurement of sTfR1 and establishing sTfR1/log ferritin ratio can be used to assess iron deficiency states [39,40]. TfR1 is the primary cellular iron gate and mediates the import of circulating transferrin-bound iron [41]. It is induced in response to iron deficiency, and its shedding from the plasma membrane gives rise to sTfR1. Thus, plasma sTfR1 concentrations reflect protein density at the cell surface and the number of cells expressing the receptor, making it a good biomarker of iron demand [42]. It may be used to differentiate “true” from “functional” iron deficiency under inflammatory conditions [43]. Nevertheless, the wide applicability of sTfR1 as a diagnostic iron biomarker is limited by the relatively high cost of the assay and the lack of international standardization [42].

Serum hepcidin can be used for assessing iron status, diagnosing iron deficiency states, and predicting responses to iron absorption from foods and supplements [44]. However, its main limitation, much like serum ferritin, is its induction by inflammation. Despite this, hepcidin may be a useful diagnostic tool in specific conditions such as chronic renal disorders or diseases of iron overload [45]. Yet, hepcidin (and sTfR1) assays have not yet become standardized tests due to harmonization challenges [40,44]. Serum hepcidin assays can vary up to 10-fold between methods and test brands due to the absence of a main reference material, a reference technique, and a commutable calibrator, making it difficult to compare data and establish a uniform reference range [46,47].

## 4. Dietary Iron Intake and the Risk for Disease

High iron stores are often viewed as a potential biohazard [15], indicating that excessive dietary iron intake increases the risk for disease. Dietary iron is found in the form of heme or inorganic (non-heme) iron [3]. Heme is primarily present in hemoglobin and myoglobin from animal food sources, while inorganic iron is found in food derived from both plants and animals [2]. Heme consists of ~10–15% of total dietary iron sources in meat-eating populations, but accounts for over 40% of assimilated iron due to its enhanced absorption [2]. Thus, it is estimated that the efficiency of inorganic iron absorption is ~10% and of heme ~25%, which is 2.5 times higher [2]. This may be related to the lipophilic nature of the heme molecule, and possibly also to lack of negative feedback regulation of heme absorption. In any case, the intestinal heme transporter remains elusive, and its identification and characterization are expected to increase our mechanistic understanding. Thus far, it is well established that dietary absorbed heme is catabolized within intestinal epithelial cells and liberated iron follows the fate of dietary inorganic iron assimilated via the divalent metal transporter 1 (DMT1). DMT1 is expressed on the apical membrane of duodenal enterocytes and is subjected to negative regulation by iron [48].

There is evidence from epidemiological studies that high dietary iron intake may predispose to diseases (Table 1). Most if not all these studies assessed dietary heme intake based on food questionnaires and data were extrapolated from meat consumption. However, it should be noted that processed red meat contains several potentially confounding substances, such as nitrate/nitrite, heterocyclic amines, polycyclic aromatic hydrocarbons, etc., which may likewise affect health outcomes. Therefore, it is not clear whether the adverse effects of high dietary heme intake can always be entirely attributed to iron. This mainly applies to heme as opposed to inorganic iron as described in an umbrella review with a total of 34 meta-analyses [49]. The intake of heme but not inorganic iron was statistically significantly associated with a modestly increased risk for type 2 diabetes mellitus (T2DM), gestational diabetes mellitus, coronary heart disease, cardiovascular disease (CVD), CVD mortality, as well as colorectal, esophageal and breast cancer [49]. It should, however, be emphasized that in most cases the quality of the evidence was low (class III or IV). In line with this notion, direct biochemical validation that dietary heme is the actual driver of the above pathological conditions is currently unavailable and awaits experimental studies. 

## 5. Iron and the Risk for Metabolic Syndrome

Metabolic syndrome is a pathologic state defined by the combined manifestation of abdominal obesity, hyperglycemia due to insulin resistance, dyslipidemia, and/or hypertension. Consequently, patients with metabolic syndrome are at greater risk of developing T2DM, CVD, non-alcoholic fatty liver disease (NAFLD), and some types of cancer. Interestingly, in a multi-ethnic, population-based cohort of 3828 participants, metabolic syndrome itself was associated with intake of heme iron [hazard ratio (HR) = 1.25 (95% CI = 0.99 to 1.56)] and zinc [HR = 1.29 (95% CI = 1.03 to 1.61)] from red meat [50]. Several clinical studies established a strong association between high body iron stores and insulin resistance [68,69,70,71], and the combination of iron overload with metabolic defects has been labeled as dysmetabolic iron overload syndrome (DIOS) [72]. Importantly, the alterations in iron metabolism appear to be multifactorial and dynamic due to an unhealthy diet combined with environmental and genetic cofactors [72]. Despite evidence that dietary iron depletion can attenuate NAFLD progression in mice [73], the benefits of phlebotomy on patients with DIOS remain controversial with a meta-analysis of 9 studies finding no significant improvement [74]. Nevertheless, the strong correlation between high dietary heme intake with T2DM and metabolic syndrome is consistent with iron-dependent effects [52,75], even though other contributing factors cannot be excluded. In fact, there are several molecular links between iron and glucose metabolism [76]. For instance, insulin stimulates ferritin synthesis thereby increasing tissue iron stores; in addition, it promotes redistribution of transferrin receptors to the cell surface, thereby stimulating iron uptake [77].

Insulin was positively associated with serum ferritin levels in the 1988–1994 cross-sectional study from the Third National Health and Nutrition Examination Survey suggesting a possible link between T2DM and iron [78]. This association has since been corroborated in smaller cohort studies in Norwegian men, subjects with excessive body weight, and adolescent girls [79,80,81]. A meta-analysis found a correlation between high serum ferritin and T2DM in 11 out of 12 studies analyzed, with an odds ratio (OR) of 1.43 (95% CI 1.29 to 1.59) [51]. The ratio of sTfR1 to serum ferritin was also inversely related to the risk of T2DM with an OR of 0.65 (95% CI 0.45 to 0.95) in participants with high serum ferritin [51]. 

A 2012 meta-analysis of 5 primary prospective cohort studies suggested a relative risk of 1.33 (95% CI 1.19 to 1.48) for T2DM in individuals with the highest vs lowest heme intake [52]. High body iron stores were likewise linked to T2DM with a relative risk of 1.70 (95% CI 1.27 to 2.27); however, total dietary or supplemental iron intake did not show any significant association [52]. Another systematic review performed in 2021 came to the same conclusion [53]. This review included data from 323,788 participants over 11 studies and found that higher heme intake was associated with a 20% increased risk of developing T2DM (95% CI 1.07 to 1.35) [53]. 

Similar results were reported in two meta-analyses on iron and gestational diabetes mellitus (GDM). Fu et al. analyzed 1025 GDM patients against 15,608 controls across 2 studies to compare the lowest and highest consumers of heme iron and found a relative risk of 1.53 (95% CI 1.17 to 2.00) for the development of GDM [54]. Kataria et al. analyzed the association between heme iron intake and GDM across 4 studies including the two studies from the previous meta-analysis and found an adjusted OR of 1.48 (95% CI 1.29 to 1.69) [55]. Both meta-analyses confirmed a strong association between GDM and high body iron stores, but not intake of total dietary iron or iron supplements [54,55]; nonetheless, the level of evidence was low, while heterogeneity among the primary data was high. Due to the limited number of studies examining heme iron intake and GDM compared to T2DM, further investigation should be performed to provide a reliable correlation.

Molecular studies in cell and mouse models have linked iron overload to insulin resistance through mechanisms involving autophagy in skeletal muscles and cardiomyocytes [82,83]. Iron loading in cell and mouse models exacerbated palmitate-induced insulin resistance, impaired glucose-stimulated insulin secretion from pancreatic β cells, and increased gluconeogenesis in the liver [84,85,86]. Conversely, iron deficiency has been associated with insulin sensitivity in rats [87,88]. In young women, correction of iron deficiency anemia decreased insulin levels [89]. Taken together, these data provide a basis to investigate molecular mechanisms underlying the increased risk for metabolic syndrome pathologies in individuals with high dietary iron intake and elevated body iron stores.

## 6. Iron and the Risk for Cardiovascular Disease

The association between iron and CVD was first proposed in 1981 from observations that the incidence of CVD was elevated in men and post-menopausal women [90]. A meta-analysis of 21 cohort studies with 292,454 participants revealed a significant association between heme iron intake and coronary heart disease incidence [56], with a relative risk of 1.57 (95% CI 1.28 to 1.94). These findings were corroborated in another meta-analysis from 6 different studies including 131,553 participants [57]. Interestingly, total iron intake, serum iron levels, and transferrin saturation were inversely correlated with coronary heart disease incidence [56]. A meta-analysis of 13 primary studies with 252,164 participants reported a relative risk of 1.07 (95% CI 1.01 to 1.14) for CVD in individuals with high dietary heme intake [58]. Another meta-analysis of 19 studies with 720,427 participants reported an association between high dietary heme intake and CVD mortality, with a relative risk of 1.19 (95% CI 1.01 to 1.39) [59]. Iron status has also been positively associated with carotid atherosclerosis in the absence of inflammation [91]. Additionally, abdominal walls from patients having suffered abdominal aortic aneurysms displayed iron accumulation compared to healthy controls with elevated expression of TfR1 [92]. On the other hand, iron deficiency is known comorbidity in patients with heart failure [93,94] and its correction with intravenous iron administration has been shown to reduce hospitalizations [95]. Taken together, these data highlight epidemiological links between iron status and CVD risk.

Animal studies have provided supportive evidence. Thus, early experiments in rabbits injected with iron dextran and fed a 0.5% cholesterol diet demonstrated greater atherosclerotic lesion development compared to the diet alone [96]. Recent work in mice suggested that non-transferrin-bound iron (NTBI), a highly redox-active form of iron that appears in the circulation primarily during conditions of iron overload, aggravates atherosclerosis [97]. Crossing apolipoprotein E knockout (apoE^–/–^) mice, an established model of atherosclerosis, with mice that express a hepcidin-resistant ferroportin mutant (Fpn^wt/C326S^) aggravated atherosclerosis via increased levels of NTBI and oxidative stress [97]. Iron loading of the heart appears to be critically important when it is in cardiomyocytes as demonstrated by the reduced survival of mice lacking ferroportin in this cell type [98]. Thus, despite having elevated cardiac iron content, mouse models of hemochromatosis exhibit minor cardiac dysfunction and develop cardiomyopathy only in response to chronic dietary or parenteral iron loading [99,100]. 

The functional importance of cardiomyocyte iron load is also emphasized by the lethal cardiomyopathy documented in mice with iron-deficient cardiomyocytes due to ablation of TfR1 [101] or expression of hepcidin-resistant Fpn^C326S^ [102]. Notably, local production of hepcidin is necessary for proper iron homeostasis in the heart [102], and a better understanding of its function and regulation is needed.

## 7. Iron and Cancer Risk

An observational study in a cohort of 309,443 adults in Taiwan identified an increased incidence of all cancers in individuals with high serum iron with a HR of 1.25 (95% CI 1.16 to 1.35), and a HR for mortality from all cancers of 1.39 (95% CI 1.23 to 1.57) [61]. Similar results were reported in a meta-analysis of 27 studies, where high serum iron correlated with a higher relative risk for breast cancer (1.22; 95% CI 1.01 to 1.47) [64]. While no association between breast cancer and total dietary iron intake or inorganic iron supplementation was documented, dietary heme intake was associated with a significant relative risk for breast cancer of 1.12 (95% CI 1.04 to 1.22) [61]. Dietary heme intake was also significantly associated with an increased relative risk for esophageal cancer (1.21; 95% CI 1.02 to 1.45) [65], and lung cancer (1.16; 95% CI 1.02 to 1.32) after adjustment for smoking history [66]. 

The meta-analysis on esophageal cancer involved 20 studies with 1,387,482 participants; interestingly total iron intake was found to be protective against esophageal cancer development (relative risk 0.85; 95% CI 0.79 to 0.92) [65]. A similar trend was also observed in the lung cancer study that involved 416,746 individuals from European countries [66]. Moreover, comparable data were obtained from meta-analyses of studies focusing on colorectal cancer [103,104]. A European prospective cohort study of 450,105 participants followed for 14.2 ± 4.0 years found a stronger positive association of colorectal cancer in the proximal vs distal colon in men with high dietary heme intake (1.11; 95% CI:1.02 to 1.20) [67]. Yet, this trend was not observed in women, and other sources of iron were not associated with colorectal cancer [67]. Conversely, phlebotomy was associated with less new visceral malignancy in a prospective multicenter randomized clinical trial of 1277 patients with peripheral arterial disease (0.65; 95% CI 0.43 to 0.97) [62]. Moreover, an analysis of 37,795 blood donors found an association with decreased risk of cancer (0.79; 95% CI 0.74 to 0.84) [63]. 

Heme may have diverse and often opposing functions in carcinogenesis [105]. In essence, above-average levels of heme intake may lead to cellular cytotoxicity, lipid peroxidation, DNA and protein oxidation, and genetic mutations promoting carcinogenesis; however, excessive heme may also protect from carcinogenesis by shifting cell metabolism towards oxidative phosphorylation and eventually inducing cell death through ferroptosis, an iron-regulated programmed cell death [105]. It should be noted that highly proliferative cancer cells have increased needs for iron and undergo reprogramming for efficient iron acquisition and retention [106]. Studies in breast, ovarian, and prostate cancer have demonstrated reduced expression of ferroportin in the tumor to increase iron retention [107,108,109]. On the other hand, TfR1 is upregulated to increase iron uptake [110,111]. In line with these data, a meta-analysis of 22 studies revealed that serum ferritin levels were significantly higher in cancer patients with a standardized mean difference of 3.07 (CI 1.96 to 4.17) [60]. 

Mouse experiments have validated clinical and epidemiological data on the role of iron as a driver of carcinogenesis and, furthermore, have provided mechanistic insights. For instance, deletion of the *Apc* gene, an important precursor in colorectal cancer development, made mice susceptible to tumorigenesis from luminal iron [112]. Other studies demonstrated that dietary heme promotes epithelial hyperplasia in mice due to oxidative stress, hyperproliferation, and reduced apoptosis of intestinal epithelial cells [113,114]. Thus, it is likely that toxicity of excess dietary heme is directly relevant to colorectal cancer. Nevertheless, since dietary heme promotes gut dysbiosis [115], which in turn may affect metabolic syndrome and other pathologies [116,117,118], it is possible that several of its adverse effects are indirect. 

Mutations predisposing to hereditary hemochromatosis, particularly the C282Y substitution in the hemochromatosis protein HFE, have been associated with an increased risk of colorectal and breast cancer providing an association between iron and these malignancies [119]. Yet the most common type of cancer associated with hereditary hemochromatosis is hepatocellular carcinoma [120], which is also a common complication of transfusion-dependent thalassemias [121]. Interestingly, hemojuvelin knockout (Hjv^–/–^) mice, a model of juvenile hereditary hemochromatosis, are predisposed to hepatocarcinogenesis by a mechanism linked to mitochondrial hyperactivity [122].

## 8. Iron and the Intestinal Microbiome

Iron is also essential for microorganisms, and excessive dietary iron intake may affect the composition of bacterial communities in the gut. Iron deprivation has been shown to cause irreversible community alterations in the human intestinal microbiome, whereas iron supplementation resulted in small person-specific shifts [123]. Changes in the microbial composition may be more relevant to specific populations or patients with increased sensitivity to such alterations. For example, the shifts in the gut microbiota from iron intake and supplementation has been proposed to influence the progression of NAFLD in obese individuals [124].

Recent studies revealed adverse effects of iron fortification or supplementation in the intestinal microbiome of children in tropical countries that are vulnerable to infectious diseases [125,126] or inflammatory bowel disease (IBD) patients [127]. In the latter, oral iron caused a decrease in *Faecalibacterium prausnitzii* and *Ruminococcus bromi*, which are important anti-inflammatory bacteria by producing butyrate [127]. IBD poses an extra challenge as it is often associated with iron-deficiency anemia in part due to gastrointestinal bleeding [128], which provides an additional source of heme that may alter microbiome composition. Experiments in mice showed that dietary heme exacerbated dextran sodium sulphate (DSS)-induced colitis and promoted the formation of adenomas [115]. Similar results were obtained in further studies involving the use of iron-fortified chow in mouse models of colitis [129,130]. 

The gut microbiota has also been shown to play a role in colorectal cancer progression in mice, by sensitizing the mucus barrier in the presence of heme [131]. Heme appears to favor the growth of *Akkermansia muciniphila*, a mucin-degrading bacterium, which may lead to disruption of the mucus barrier [132]. Nevertheless, there is evidence suggesting that iron supplementation may be beneficial for the microbiome depending on formulation [133,134]. Additional studies would be needed to evaluate the impact of dietary or various forms of supplementary iron on the intestinal microbiome in the general population and in specific patient groups.

## 9. Conclusions

**Figure 4:**
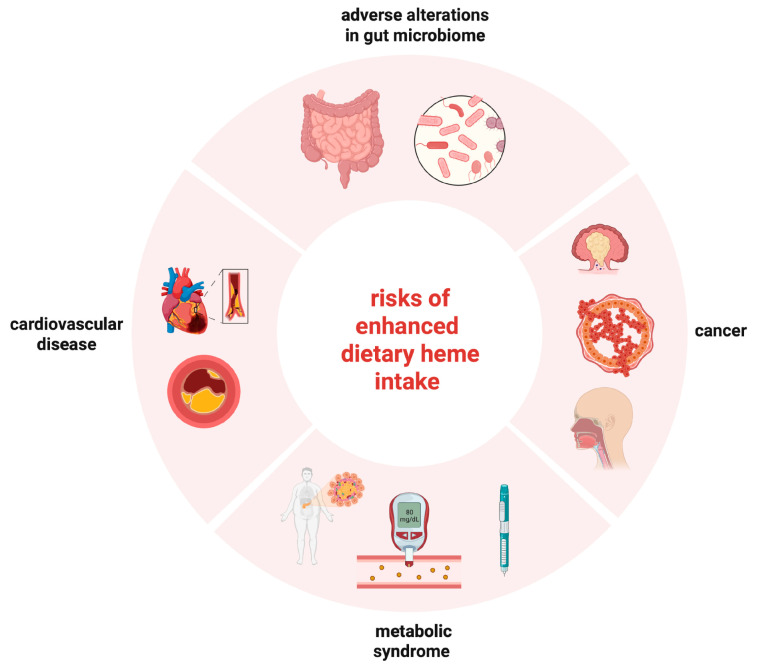
Disease risks associated with enhanced dietary heme intake.

There is evidence from epidemiological studies that high dietary heme intake via meat consumption increases the risk for adverse health effects (Figure 4), particularly T2DM, GDM, coronary heart disease, CVD, and some cancers (such as colorectal, esophageal, and breast cancer). These effects can be at least partially attributed to excessive iron accumulation due to the increased bioavailability of heme iron. However, a contribution of confounding factors present in meat products is also possible, while in most cases the HR or OR values were relatively low (below 2), indicating weak correlations. Moreover, the quality of the evidence was likewise low. These potential limitations may be offset by the large datasets obtained from study cohorts including high numbers of participants and patients (Table 1).

It is reasonable to assume that iron-dependent adverse effects of heme are more evident in conditions that are also associated with high body iron stores, such as metabolic syndrome pathologies, GDM, and some cancers, especially colorectal. Nevertheless, a clear causal relationship between dietary heme iron intake and disease risk has not been established. Thus, further experimental studies are needed to corroborate epidemiological data and explore mechanisms underlying the pathogenicity of high dietary heme iron. 

There is no evidence that dietary intake of inorganic iron, even from iron-fortified foods or supplements, contributes to disease burden in the general population. Regardless, it will be important to understand the impact of dietary iron on the intestinal microbiome, especially in the context of inflammatory and infectious diseases.

## Figures and Tables

**Table 1:** Pathologies with significant iron-related associations.

Pathology	Risk Factor	#of Studies	# of Patients (# of Controls or Total Participants)	Risk	95% CI	Reference
Metabolic Syndrome	Heme intake	1	3828	1.25	0.99–1.56	de Oliveira et al. [50]
Type 2 Diabetes Mellitus	Serum ferritin	12	6516 (43,120)	1.43	1.29–1.59	Liu et al. [51]
	sTfR/Serum ferritin	12	6516 (43,120)	0.65	0.45–0.95	Liu et al. [51]
	Heme intake	5	9269 (192,635)	1.33	1.19–1.48	Bao et al. [52]
	Body iron stores	5	9269 (192,635)	1.7	1.27–2.27	Bao et al. [52]
	Heme intake	11	323,788	1.2	1.07–1.35	Shahinfar et al. [53]
Gestational Diabetes Mellitus	Heme intake	2	1025 (15,608)	1.53	1.17–2.00	Fu et al. [54]
	Heme intake	4	1513 (25,647)	1.48	1.29–1.69	Kataria et al. [55]
Coronary Heart Disease	Heme intake	21	12,721 (292,454)	1.57	1.28–1.94	Hunnicutt et al. [56]
	Heme intake	6	2459 (131,553)	1.31	1.04–1.67	Yang et al. [57]
Cardiovascular Disease	Heme intake	13	15,040 (252,164)	1.07	1.01–1.14	Fang et al. [58]
Cardiovascular Disease (Mortality)	Heme intake	19	46,045 (720,427)	1.19	1.01–1.39	Han et al. [59]
All Cancers	Serum ferritin	22	1080 (1157)	3.07	1.96–4.17	Ramirez-Carmona et al. [60]
	Serum iron	1	8060 (309,443)	1.25	1.16–1.35	Wen et al. [61]
	Phlebotomy	1	1277	0.65	0.43–0.97	Zacharski et al. [62]
	Blood donation	1	1152 (37,795)	0.79	0.74–0.84	Merk et al. [63]
Breast Cancer	Serum iron	27	39,453 (885,680)	1.22	1.01–1.47	Chang et al. [64]
	Heme intake	27	39,453 (885,680)	1.12	1.04–1.22	Chang et al. [64]
Esophageal Cancer	Heme intake	20	4855 (1,387,482)	1.21	1.02–1.45	Ma et al. [65]
	Total iron	20	4855 (1,387,482)	0.85	0.79–0.92	Ma et al. [65]
Lung Cancer	Heme intake	1	3731 (416,746)	1.16	1.02–1.32	Ward et al. [66]
Colorectal Cancer	Heme intake	1	6162 (450,105)	1.11 (in men)	1.02–1.20	Aglago et al. [67]

## Data Availability

Data sharing not applicable.
